# Theory of Mind Mediates the Association Between Autistic Traits and Social Isolation in Middle‐Aged and Older Adults

**DOI:** 10.1002/aur.70036

**Published:** 2025-04-04

**Authors:** Gloria Hei Man Lo, Clemie Dale, Francesca Happé, Gavin R. Stewart

**Affiliations:** ^1^ Social, Genetic and Developmental Psychiatry Centre, Institute of Psychiatry, Psychology & Neuroscience King's College London London UK

**Keywords:** aging, autism, autistic traits, midlife, older age, social connectedness, social isolation, theory of mind

## Abstract

Social isolation has detrimental effects on wellbeing. While isolation can occur at any age, its prevalence has been found to increase in older adulthood. Populations with social functioning differences, such as autistic people, have also been found to be at particular risk of isolation across the lifespan, including in older age. Despite the widespread impacts of isolation, little is known about the underlying factors that may contribute to social isolation in autistic people and the general populations. While social isolation has been linked to autistic traits and theory of mind (ToM), no study has yet considered their inter‐relationship. Taking a dimensional approach to autistic traits, this study examined the association between autistic traits (assessed by the AQ‐10), ToM (CarToM and Frith–Happé Triangles) and social isolation (Lubben Social Connectedness Scale) among 111 adults (*n* = 53 autistic, 58 non‐autistic), aged 40–86 years. The study also assessed the putative mediating role of ToM in the association between autistic traits and isolation. Pearson correlational analyses showed middle‐aged and older adults with higher social connectedness reported fewer autistic traits and showed better performance in ToM tasks, even when accounting for the effect of age and mental health symptoms. Mediation analyses suggested the association between autistic traits and social isolation was partially mediated by ToM when age and mental health symptoms were accounted for. These findings suggest one possible mechanism for the experience of social isolation. Additionally, the findings highlight that autistic people and people with high autistic traits may be particularly susceptible to social isolation in midlife and older age, and may benefit from additional support and possible interventions to maintain desired levels of social connectedness in later life.

**Summary**

- While social isolation can occur at any age, older people are at a greater risk of becoming isolated.
- Autistic people, who often have social functioning differences, are also at a higher risk of isolation across the lifespan, including in older age.
- Despite this, little is known about the factors that influence social isolation in middle‐aged and older autistic adults.
- This study explored how middle‐aged and older adults' social connectedness, autistic traits, and ability to infer mental states (e.g., beliefs, intentions) are related.
- We found that individuals who are more socially connected with family and friends show fewer autistic traits and a better ability to infer mental states.
- Additionally, individuals' ability to infer mental states seems to play an important role in affecting their social connectedness, especially among autistic adults in midlife and older age.

## 1 Introduction

Social isolation is a prevalent yet neglected health concern. While associated with poorer wellbeing across the lifespan, it is particularly impactful in older age (Holt‐Lunstad 2024). The prevalence of social isolation among older adults has greatly increased, ranging from 7% to 24% in the previous decades (Chen and Schulz 2016), and upwards of 31% during/since the COVID‐19 pandemic (Su et al. 2022; Malani et al. 2023). Social isolation is often differentiated into objective isolation, indicated by the deprivation of social connectedness (such as social contact or relationships), and subjective isolation, such as feelings of loneliness and longing for social connections (Cornwell and Waite 2009; Valtorta and Hanratty 2012). While distinct, these types of social isolation are closely related (Petersen et al. 2015), and are both associated with adverse health outcomes in older adulthood, including increased rates of depression and anxiety (Courtin and Knapp 2015; Taylor et al. 2016), lower quality of life (Newman‐Norlund et al. 2022), poorer cognitive function (Lara et al. 2019; Cardona and Andrés 2023), dementia (Penninkilampi et al. 2018), suicidal ideation (Chang et al. 2017; Heuser and Howe 2019) and mortality (Steptoe et al. 2013; Naito et al. 2021). Such findings raise concerns about the impact of prolonged social isolation, with isolation and loneliness being declared as pressing global health threats (Office of the Surgeon General 2023). In response, the World Health Organization (WHO) has launched a commission to promote social connection and has called for research to investigate potential mechanisms underlying the risk of social isolation experienced by older adults (WHO 2023; O'Rourke 2024).

While these calls for investigation have been directed towards the general population, people on the autism spectrum have been found to experience high rates of social isolation (Stice and Lavner 2018; Grace et al. 2022), including in older age (Stewart et al. 2024). Socio‐communicative differences, a core feature of autism (American Psychiatric Association 2013), as well as associated social stigma and lack of understanding by neurotypical society (Sasson et al. 2017), may be key factors that lead autistic people to become isolated.

To date, there is a scarcity of research that focuses on the experiences of autistic people in older age (Mason et al. 2022). To the authors' knowledge, only four studies have examined objective social isolation among autistic adults in midlife and older age. Hickey et al. (2017) conducted semi‐structured interviews with 13 autistic participants aged 51 to 71 years (mean age = 60) found most participants perceived themselves as socially isolated. Viner et al. (2024) (*n* = 16) and Francis et al. (2025) (*n* = 15) also found similar sentiments about social isolation in their qualitative studies of autistic adults in midlife and older age (ranges 40–63 and 50–78, respectively). Finally, a quantitative study by Stewart et al. (2024) examined social isolation and loneliness among 265 autistic adults and 167 non‐autistic comparisons aged 40 to 93 years (mean age = 60). Increased social isolation and loneliness were observed to be strongly associated with age, with the autistic group reported being less socially connected with family or friends than comparisons. In addition, significantly higher depression and anxiety scores were reported among autistic individuals and those with higher loneliness and social isolation. Taken together, these findings suggest that autistic individuals in midlife and older age, particularly older autistic adults, may be highly susceptible to experiencing social isolation. This highlights the need to support autistic people in maintaining desired social connectedness, which may help to address a broad range of difficulties reported by aging autistic people, such as poor mental health.

A potential mechanism that could be explored in why autistic people experience high rates of social isolation is the differences in aspects of social cognition, specifically theory of mind (ToM), that underpin successful social interactions (Baron‐Cohen et al. 1985; Happé 2015; Mazza et al. 2017). ToM is the ability to infer the mental states of self and others, including understanding that others may have different perspectives (Happé et al. 1998; Happé 2015). Automatic, intuitive and effortless ToM being a core difference between cognition in neurotypical and autistic populations has long been debated (e.g., the “double empathy problem” account, where the social difficulties experienced by autistic people arise due to a “mismatch” between communication styles with non‐autistic people; Milton 2012), with recent papers critiquing the way in which ToM is conceptualized and studied in autistic populations (see Gernsbacher and Yergeau (2019) for review), and others cautioning the conceptualisation of double empathy and lack of empirical evidence that underpins the theory (see Livingston et al. 2024). However, many studies have reported widely replicated findings of lower performance in ToM tasks by autistic (e.g., Livingston et al. 2021) and high autistic trait people (e.g., Sasson et al. 2012) when compared to non‐autistic/low autistic trait comparisons. Like other cognitive domains, age differences in ToM have also been found in the general population and those on the autism spectrum (e.g., Stewart et al. 2019; De Lillo et al. 2023). However, a similar pattern of results has been found in studies involving older adults (*n* = 47, aged 60–91 years); participants with high autistic traits scored lower in ToM tasks than comparisons with low autistic traits (Stewart et al. 2019). As such, consistent findings from studies suggest autistic traits as a reliable variable in predicting ToM performance across the lifespan.

When examining potential relationships between ToM and social isolation in the general population, studies focusing on older adult samples have reported associations between better ToM ability and being well socially connected. A study by Lecce et al. (2015), which examined 53 older adults (aged 60–85 years; mean age = 68 years), observed that individuals with better ToM understanding were more likely to report being well socially connected to their friends. These findings were later replicated by Lecce et al. (2018) in another study (*n* = 71; aged 60–79; mean age = 68 years) and further supported by De Lillo et al. (2023) who observed increasing loneliness with decreasing ToM task performance among 98 older adults (aged 60–86; mean age = 70 years).

Although several studies have found evidence for the relationship between ToM ability, autistic traits, and social connectedness, to the authors' knowledge, no study has yet examined the interlinked associations. As such, the present study sought to contribute to the emerging literature in understanding the underlying factors of social isolation among older autistic adults, by studying the relationship in between. When examining this relationship, age is an important factor to be accounted for due to associations with social isolation (e.g., De Lillo et al. 2023; Stewart et al. 2024) and ToM, in high autistic trait and general populations (Stewart et al. 2019; De Lillo et al. 2023). With high rates of autistic and high autistic trait individuals experiencing anxiety and depression (Wallace et al. 2016; Hollocks et al. 2018; Stewart et al. 2021), as well as reported associations with social isolation (Grace et al. 2022; Stewart et al. 2024), mental health is another important factor to be accounted for. Given the documented under‐diagnosis of autism in adults over 50 years (O'Nions et al. 2023), we took a dimensional approach to autistic traits, supported by behavioral and genetic evidence of a continuum of traits from diagnosed through subclinical and individual differences (Tick et al. 2016). This study will examine whether performance on two similar but different computerized ToM tasks (one selected for being widely used and not having language demands, the other for low memory load) mediates the relationship between autistic traits and social isolation in a sample of middle‐aged and older adults.

In the present study, it is hypothesized that: (1) associations will be found between social isolation (inferred from having low social connectedness to family and friends), high autistic traits, and lower performance scores in the two ToM tasks, including when (2) accounting for age and symptoms of poor mental health. Additionally, (3) the relationship between autistic traits and social isolation will be mediated by ToM performance, including when accounting for age and symptoms of poor mental health.

## 2 Methods

### 2.1 Study Design

The present study examines cross‐sectional data collected between April and June 2023. The purpose of this study was to explore the relationship between social connectedness/isolation and ToM and autistic traits. This study received ethical approval from the King's College London Health Faculties (Blue) Research Ethics Subcommittee (HR/DP‐22/23‐35508). All participants provided informed consent. Participation was anonymous and no identifiable information was collected. Participants could choose to be entered into a gift voucher draw for one of ten £50 gift vouchers.

Prior to the development of this study, an in‐person focus group involving six older autistic adults (four women, two men; all aged 70 years and older) was conducted by GRS to refine the aims and scope of this project. The group members also trialed the study to ensure ease of access for older participants. Each member was paid a £75 gift voucher for their time and contribution to the project, aligning with NIHR INVOLVE payment for half a day's activity. An overview of the focus group is provided in Supporting Information A.

Participants were recruited through several channels, including study adverts on social media (e.g., Twitter/X and Reddit), through the ReSpect Lab participant database, and other older adult community groups. Inclusion criteria for the study were: (1) being 40 years or older, (2) having access to an internet‐connected device, and (3) being able to read and type in English. No specific exclusion criteria were stated. Participants accessed the survey via Qualtrics. Prior to beginning the survey, a full information sheet and consent form were presented that detailed the aims and objectives of the study. Participants were then presented with a series of standardized questionnaires that explored a variety of topics, including (1) demographic factors, (2) autistic traits, (3) social connectedness, and (4) symptoms of poor mental health. Participants then completed two ToM tasks that had been designed for online use. Once completed, participants were presented with a reminder of the study information sheet and the opportunity to enter their email into a gift voucher draw.

### 2.2 Participants

In total, 364 complete responses were recorded. Upon comprehensive screening for spam participants (an issue highlighted by Pellicano et al. (2024)), 253/364 responses (69%) were identified as being suspicious. This screening process involved two members of the research team reviewing each survey response, with suspicious responses having one or more of the following: (1) very short completion times/very low completion rates of questions, which suggest the participant clicked through the survey (approx. 20% completed in under 10 min); (2) nonsensical responses to open‐text boxes (approx. 30% repeated question instructions designed to trick bots or used non‐English responses); (3) low CAPTCHA scores, which indicate a high probability of bot entries (approx. 20% scored under suggested threshold of 0.5).

The final total of 111 responses passed our thorough quality control checks. Participants were aged 40 to 86 years (*M* = 58.26, SD = 11.84). In total, 79.3% of the participants were white. Additionally, 75.7% of the sample was recruited from the United Kingdom, 8.1% from Europe, 6.3% from Canada, 5.4% from the United States and 4.5% from Asia. Of the 111 participants, 35 (31%) reported having an autism diagnosis from a medical professional, with an additional 18 (16%) being self‐identified as autistic. See Table 1 for demographic details of the whole sample.

**TABLE 1:** Demographic characteristics of the whole sample.

	Total (*n* = 111)
Age, years	
*M* (SD)	58.26 (11.84)
Min	40
Max	86
Gender, *n* (%)	
Men	48 (43.2%)
Women	62 (55.9%)
Nonbinary	1 (0.9%)
Ethnicity, *n* (%)	
White	88 (79.3%)
Asian	12 (10.8%)
Black	4 (3.6%)
Mixed or multiple ethnicities	4 (3.6%)
Middle Eastern or Arab	3 (2.7%)
Education, *n* (%)	
Below degree level	62 (55.9%)
Degree or above	49 (44.1%)
Household members, *n* (%)	
Live alone	47 (42.3%)
2	41 (36.9%)
3	10 (9%)
4	9 (8.1%)
5	4 (3.6%)
Employment, *n* (%)	
Full‐time employment	47 (42.3)
Part‐time employment	4 (3.6%)
Volunteer/unpaid	4 (3.6%)
Career	1 (0.9%)
Unemployed	6 (5.4%)
Unable to work due to health	9 (8.1%)
Retired	38 (34.2%)
Self‐employed	8 (7.2%)
In supported full‐time employment	1 (0.9%)
Autism diagnosis, *n* (%)	
Received diagnosis	35 (31.5%)
Self‐identified as autistic	18 (16.2%)
No diagnosis/not identified as autistic	58 (52.3%)

*Note*: Autism diagnosis based on self‐report.

### 2.3 Materials

#### 2.3.1 Demographic Characteristics

Participants provided detailed demographic information, including age, gender, education level, country of residency, education qualification, employment status, living situation, and whether they have an autism diagnosis or self‐identify as autistic.

#### 2.3.2 Self‐Report Measures

##### 2.3.2.1 Autistic Traits

The 10‐item Autism Spectrum Quotient (AQ‐10; Allison et al. 2012) was used to measure autistic traits. The AQ‐10 uses a 4‐point Likert scale, ranging from “*Strongly disagree*” to “*Strongly agree*.” When scored, the “agree” and “disagree” responses are collapsed, with scores ranging from 0 to 10. Scores of ≥ 6 suggest an individual may be autistic and further formal clinical investigation is warranted. This threshold is reported to have a sensitivity of 79.9% and specificity of 87.3% for diagnosed autism (Booth et al. 2013). In the present study, we used a dimensional approach for autistic traits, treating the AQ‐10 score as a continuous variable. The internal consistency of the AQ‐10 was excellent in the current study (Cronbach's *α* = 0.95).

##### 2.3.2.2 Social Connectedness and Isolation

The six‐item Lubben Social Network Scale (LSNS‐6) was used to measure social connectedness with family and friends (Lubben et al. 2006). The LSNS‐6 has two subscales that explore social integration and support access availability from (a) family members and (b) friends. The LSNS‐6 uses a six‐point scale, ranging from “0 = *none*” to “5 = *nine or more*” connections. Scores are totalled for each subscale, as well as an overall combined total score, with total scores ranging from 0 to 30. High scores indicate good social integration and support access, while low scores indicate social isolation and low support access. The LSNS‐6 has been widely used in older adult populations and has high internal consistency and stable factor structure (Lubben et al. 2006). The LSNS‐6 has also demonstrated good cross‐cultural validity (Chang et al. 2018) and very good/excellent psychometric properties in middle‐aged and older autistic populations (Stewart et al. 2024). In the current study, the internal consistency of the LSNS‐6 was rated very good (Cronbach's *α* = 0.87).

##### 2.3.2.3 Symptoms of Poor Mental Health

The eight‐item Patient Health Questionnaire (PHQ‐8; Kroenke et al. 2009) and the seven‐item Generalized Anxiety Disorder questionnaire (GAD‐7; Spitzer et al. 2006) were used to assess current symptoms of depression and anxiety, respectively. These measure the frequency of hallmark symptoms over the past 2 weeks using a 4‐point response scale of “0 = *not at all*” to “3 = *nearly every day*”. Scores in the PHQ‐8 range 0–24 and GAD‐7 range 0–21, with higher scores indicating greater symptom severity. Both measures have been widely used in middle‐aged and older populations and have been found to have excellent psychometric properties. In the present study, the internal consistency of the PHQ‐8 and GAD‐7 were very good (Cronbach's *α* = 0.82 and 0.85, respectively).

#### 2.3.3 ToM Tasks

##### 2.3.3.1 Cartoon Theory of Mind (CarToM) Task

The first ToM task used in this study is the recently developed CarToM task (Livingston et al. 2023). Participants are presented with 31 pairs of black and white line art cartoons, of which three are practice trials, 14 have a humorous element related to ToM, and 14 have a humorous element related to non‐ToM. The ToM cartoons require ToM reasoning to understand the underlying humor (e.g., the character in the cartoon has a false belief), while the non‐ToM cartoons require physical reasoning to understand the humor (e.g., physical impossibility). The 28 ToM/non‐ToM pairs are randomized in presentation, with a holding screen between each trial to ensure the participant's cursor returns to the center of the screen. See Supporting Information B for example pairs of cartoons taken from Livingston et al. (2023).

During the CarToM task, participants are instructed to select from the pair the cartoon that they perceived as more humorous as quickly and accurately as possible. Each trial is scored on accuracy (0/1) and selection reaction time (msec). Percentage accuracy and mean reaction times for correct trials were calculated for the ToM and non‐ToM conditions separately. Scores were then integrated using a linear integrated speed–accuracy score (LISAS) method (Vandierendonck 2017, see Livingston et al. 2023). Overall, a higher LISAS score reflects poorer task performance and thus poorer ToM. Internal reliabilities were reported to be acceptable in both ToM and non‐ToM conditions among autistic and non‐autistic adults (Livingston et al. 2023). In the present study, the internal consistency of the CarToM ToM and non‐ToM scales was acceptable (Cronbach's *α* = 0.70 and 0.65, respectively).

##### 2.3.3.2 Frith–Happé Triangles Animations

The second ToM task used in this study was the widely used Frith–Happé Triangles Animations task (FHTA task; Abell et al. 2000; Castelli et al. 2002). The FHTA task involves six short animated clips involving two moving triangles. The task is designed to measure the participant's ability to attribute mental states to the triangles that performed specific actions in each clip. There are two goal‐directed animations and four ToM‐based animations. In the present study, only responses to the ToM movement animations were further analyzed. The ToM scenarios involve the triangles performing actions related to coaxing, surprising, mocking, and seducing.

Responses were collected via an open text box, with scoring involving a qualitative assessment of the participant's description of each animation. Responses are scored for appropriateness (0–2, max score = 8), intentionality (0–5, max score = 20), and whether mental states are described (0–1, max score = 4). A higher score represents better performance in each subscale. In the present study, the three subscale scores were combined into an overall composite score (0–32). Prior to scoring, GRS provided training to GHML and CD in coding and scoring the open‐text responses. The three authors coded/scored 20% of the responses during the training and calibration sessions, with GHML and CD coding the remaining 80% of responses independently. Inter‐rater reliability between the coders was very good to excellent (Appropriateness Intraclass Correlation Coefficient (ICC) = 0.89, Intentionality ICC = 0.85, Mental State Speech ICC = 0.80; Composite ICC = 0.94). In the present study, the internal consistency of the FHTA scores were very good (Cronbach's *α* = 0.88).

### 2.4 Statistical Analyses

All statistical analyses were performed in IBM SPSS Statistics (Version 29). All analyses were conducted on the whole sample (*n* = 111). Statistical power and sample size were calculated using G*Power prior to data collection, and assumptions were tested prior to analyses (i.e., normality of data, linearity of data, examination for outliers, and checks for appropriateness for mediation). Pearson correlations were used to explore the associations between social connectedness, autistic traits, and ToM performance, with partial correlations being used to control for the effect of age and symptoms of depression and anxiety. Mediation analyses were performed using PROCESS for SPSS (Version 4.2) to examine the mediating effect of ToM (separate models for CarToM and FHTA) on the association between autistic traits and social connectedness while controlling for age, symptoms of depression and anxiety. This study and analysis plan were not pre‐registered.

The False Discovery Rate (FDR; Benjamini and Hochberg 1995) with an initial critical value of 0.05 was used in the present study to control for multiple comparisons. All significant associations remained once FDR was conducted.

## 3 Results

### 3.1 Associations Between Social Connectedness, Autistic Traits, and ToM Performance

Significant associations were found between social connectedness, autistic traits, and ToM task performance. Lower social connectedness (representing greater social isolation) was associated with higher autistic traits and poorer ToM task performance (represented by a higher LISAS score in CarToM, and lower composite score in FHTA task). See Table 2 for correlation matrix.

**TABLE 2:** Descriptive statistics and correlations between variables.

Variables	*M* (SD)	Social connectedness	Autistic traits	CarToM LISAS	FHTA composite	Age	Depressive symptoms	Anxiety symptoms
Social connectedness (scores 0–30)	8.93 (5.32)	1						
Autistic traits (scores 0–10)	4.77 (4.21)	−0.43***	1					
CarToM LISAS	23324.88 (10468.46)	−0.51***	0.28**	1				
FHTA composite	19.98 (7.97)	0.53***	−0.58***	−0.35***	1			
Age	58.26 (11.84)	−0.20*	−0.14	0.64***	−0.02	1		
Depressive symptoms (scores 0–27)	8.73 (5.58)	−0.37***	0.65***	0.20*	−0.26**	−0.14	1	
Anxiety symptoms (Scores 0–21)	8.11 (5.44)	−0.35***	0.61***	0.16	−0.28**	−0.25**	0.82***	1

*Note*: Higher social connectedness = connected with numerous family and friends; Higher CarToM LISAS = poorer performance; Higher FHTA = better performance.

Abbreviations: CarToM, Cartoon Theory of Mind; FHTA, Frith–Happé Triangles Animations; LISAS, linear integrated speed—accuracy score.

* *p* < 0.05.

** *p* < 0.01.

*** *p* < 0.001.

#### 3.1.1 Associations With Age and Symptoms of Poor Mental Health

Older age was associated with lower social connectedness and with poorer performance in the CarToM (represented by a higher LISAS score). Depression and anxiety were associated with each other and also with lower social connectedness, higher autistic traits, and poorer performance in the FHTA task. Depression was positively associated with CarToM LISAS score (i.e., lower ToM performance), and anxiety was negatively associated with age. See Table 2 for correlation matrix.

#### 3.1.2 Covariate Controlled Associations

When controlling for the influence of (1) age, (2) symptoms of depression, and (3) symptoms of anxiety, a similar pattern of results was found between social connectedness, autistic traits, and ToM task performance. Fisher's *r*‐to‐*z* transformation indicated no significant difference in correlation coefficients between unadjusted and controlled analyses (see Supporting Information C for correlations matrices).

### 3.2 Examining the Mediating Role of ToM on the Association Between Autistic Traits and Social Connectedness

Drawing data from the two ToM tasks respectively, two simple mediation models were examined to explore the mediating effect of ToM on the relationship between autistic traits and social connectedness while controlling for the effect of age.

#### 3.2.1 Mediation Model 1—CarToM Task

The first regression analysis showed that the effect of autistic traits on ToM LISAS score was significant (*β* = 0.38, *p* < 0.001), that is, higher autistic traits predicted poorer ToM performance. As autistic traits increased, individuals reported higher ToM LISAS scores. The second regression analysis showed that the effect of ToM LISAS score on the level of social connectedness was also significant (*β* = −0.46, *p* < 0.001), that is, poorer ToM performance predicted lower social connectedness.

**FIGURE 1:**
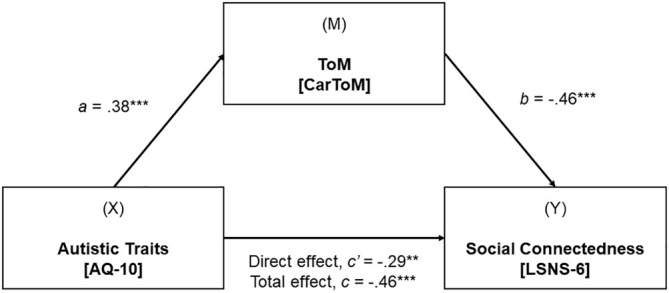
Simple mediation model between autistic traits, ToM task performance (CarToM LISAS) and social connectedness while controlling age. *Note: X* = independent variable, *M* = mediating variable, and *Y* = dependent variable. Path a = standardized regression coefficient representing the relationship between autistic traits and CarToM LISAS score. Path b = standardized regression coefficient representing the relationship between CarToM LISAS score on the level of social connectedness. Path c′ = standardized regression coefficient for a direct effect of autistic traits on the level of social connectedness. Path c = standardized regression coefficient for the total effect of autistic traits on the level of social connectedness through the mediator. *p < 0.05, **p < 0.01, ***p < 0.001.

The total effect and direct effect of autistic traits on the level of social connectedness were significant (total: *β* = −0.46, *p* < 0.001; direct: *β* = −0.29, *p* < 0.01). Following a standard bootstrapping method (Hayes and Rockwood 2017), the indirect effect of autistic traits on social connectedness through ToM LISAS score was also statistically significant (*β* = −0.17, 95% CI −0.30, −0.08). The bootstrapped confidence intervals for the standardized indirect effect of autistic traits on levels of social connectedness through ToM LISAS score did not straddle zero. Thus, the mediation model demonstrated that the association between autistic traits and social connectedness was partially mediated by ToM (when measured by the CarToM LISAS score). See Figure 1 for this mediation model.

#### 3.2.2 Mediation Model 2—FHTA Task

The first regression analysis showed that the effect of autistic traits on FHTA composite score was significant (*β* = −0.60, *p* < 0.001), that is, higher autistic traits predicted poorer ToM performance. The second regression analysis showed that the effect of FHTA composite score on the level of social connectedness was also significant (*β* = 0.38, *p* < 0.001), that is, poorer ToM performance predicted lower social connectedness.

**FIGURE 2:**
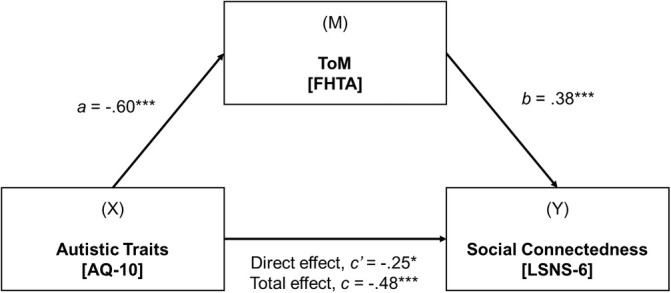
Simple mediation model between autistic traits, ToM task performance (FHTA composite) and social connectedness while controlling for age. *Note: X* = independent variable, *M* = mediating variable, and *Y* = dependent variable. Path a = standardized regression coefficient representing the relationship between autistic traits and FHTA composite score. Path b = standardized regression coefficient representing the relationship between FHTA composite score on the level of social connectedness. Path c′ = standardized regression coefficient for a direct effect of autistic traits on the level of social connectedness. Path c = standardized regression coefficient for the total effect of autistic traits on the level of social connectedness through the mediator. *p < 0.05, **p < 0.01, ***p < 0.001.

The total effect and direct effect of autistic traits on the level of social connectedness were significant (total: *β* = −0.48, *p* < 0.001; direct: *β* = −0.25, *p* < 0.05). Following a standard bootstrapping method (Hayes and Rockwood 2017), the indirect effect of autistic traits on social connectedness through FHTA composite score was also statistically significant (β = −0.23, 95% CI −0.38, −0.11). The bootstrapped confidence intervals for the standardized indirect effect of autistic traits on levels of social connectedness, through FHTA composite score, did not straddle zero. Thus, the mediation model demonstrated that the association between autistic traits and social connectedness was partially mediated by ToM (measured by the FHTA composite score). See Figure 2 for the mediation model.

When accounting for depressive and anxiety symptoms as covariates, results suggest that the association between autistic traits and social connectedness was fully mediated by ToM in both CarToM and FHTA tasks (see Supporting Information D for mediation models).

## 4 Discussion

The present study is the first to examine the mediating relationship of ToM on the association between autistic traits and social connectedness/isolation in middle‐aged and older adults. As hypothesized, middle‐aged and older adults who are well socially connected were found to have lower autistic traits and better ToM ability (measured by two established computerized tasks). The relationships between social connectedness, autistic traits, and ToM persisted when accounting for the effect of age and poor mental health symptoms. These findings suggest that middle‐aged and older people who are autistic or who have high autistic traits could potentially be more susceptible to social isolation than their non‐autistic or low autistic trait peers. Furthermore, our age‐controlled results—which aligned with hypotheses based on the existing literature—indicated that ToM ability partially mediated the association between autistic traits and social connectedness in our middle‐aged and older adult sample. Additionally, a mediation model controlling for anxiety and depression suggested ToM fully mediated the association. Taken together, these findings highlight the importance of ToM ability in the relationship between autistic traits and social connectedness, and that individual differences in ToM ability should be considered when developing support to reduce social isolation in autistic and high autistic trait populations in midlife and older age. Such supports might include, for example, facilitating activities that require less perspective taking, offering structured social activities centered around shared interests, and spaces where autistic people can engage with other autistic people.

The first key finding of the current study is that it replicates and extends the existing literature that has found social isolation (i.e., low social connectedness) to be associated separately with higher autistic traits (Stice and Lavner 2018; Grace et al. 2022), and with poorer ToM task performance (Lecce et al. 2015, 2018). Our study adds to this literature by examining the relationship between these three closely interlinked variables, including the identification of ToM as a possible cognitive underpinning contributing to social isolation.

To the authors' knowledge, only two studies (one quantitative, one qualitative) have examined the experience of social isolation in older adults who are autistic or have high autistic traits (i.e., Stewart et al. 2024; Hickey et al. 2017). Given the present study employs a dimensional approach to autistic traits instead of autism group comparison, the present study findings may not be directly comparable to the previous quantitative study by Stewart et al. (2024). Despite this, our findings broadly aligned with this study's, which found autistic adults aged 40 or above reported significantly lower social connectedness than non‐autistic peers. Additionally, our study also observed the same age‐related association, where age was negatively associated with social connectedness (i.e., older age, lower social connectedness). Taken together, these findings highlight the importance of identifying factors associated with social isolation in autistic/high autistic trait populations, to ensure support systems are designed in autism‐friendly ways to mitigate the risk of social isolation in these populations as they age.

Our second key finding is the identification of a possible cognitive mechanism underpinning the risk of social isolation in autistic/high autistic trait populations. While the relationships between autistic traits, ToM, and social isolation have previously been studied separately (e.g., Lecce et al. 2015, 2018; Stice and Lavner 2018; Stewart et al. 2019, 2024), the current study is the first to consider their interlinked associations in understanding a possible mechanism for the experience of social isolation among older autistic people. Our results suggest middle‐aged and older adults' ToM ability partially accounts for the association between higher autistic traits and lower social connectedness. When accounting for symptoms of depression and anxiety as additional covariates in a further mediation analysis, ToM ability was found to fully mediate the association across both ToM tasks. This change in the mediating role of ToM suggests that the original apparent direct effect of autistic traits on social connectedness (controlling for age) reflected in part by the association of poor mental health with autism/high autistic traits (e.g., Hollocks et al. 2018). Taken together, the findings of this study connect the results of several studies that have reported separate associations between autistic traits, ToM, and social isolation, highlighting that ToM ability may play an important role as a potential cognitive mechanism in their interlinked relationship.

When contextualizing these findings, it is important to consider the two ToM tasks used in this study. The FHTA task is a longstanding and widely used animated computerized task that includes a range of ToM and non‐ToM related scenarios depicting anthropomorphized shapes. However, the CarToM is a newer measure that depicts cartoon people in various humorous scenarios that involve ToM and non‐ToM reasoning, with the benefit of minimal memory demands. These two tasks could be criticized for low ecological validity, as they do not show real people in everyday scenarios (as discussed by Long et al. (2025) in a recent theoretical note on ToM measures), which may limit their ability to measure real world ToM ability during social interactions. Additionally, performance in these two tasks was found to correlate only modestly in the current study, indicating they may be examining similar but distinct socio‐cognitive processes. Another consideration is that while performance in the FHTA was not found to be associated with age, lower CarToM performance was associated with older age. This may be because the CarToM LISAS score combined ToM accuracy and reaction time/motor speed response; reaction time generally increases in older age. To account for this association, we opted to control for age in our analyses.

There are several important implications of our findings, particularly when creating or adapting interventions to mitigate the risk of social isolation in older populations. A widely replicated finding (including by the current study) is that social isolation is often associated with older age. The mediation analyses in the current study suggest that ToM may be an important cognitive mechanism in the experience of social isolation for autistic/high autistic trait people as they age. Therefore, interventions may need to be adapted to account for an individual's ToM abilities to increase the efficacy of the support. Additional adaptations may be needed to ‘match’ neurotypes in interventions like social prescribing, as discussed in theories like the “Double Empathy Problem.” Indeed, poorer performance by those with high autistic traits on ToM tasks in this study should be interpreted in light of criticisms that ToM tasks are designed and normed with neurotypical social cognition and performance in mind (Long et al. 2025). It may be related to social isolation because these older individuals are having to function in a neurotypical world and make social connections with mainly non‐autistic people. With ToM in mind, these adaptations could mitigate the risk of social isolation and the negative factors associated with it (e.g., Courtin and Knapp 2015). Moreover, the amplified mediating role of ToM after taking account of the depression and anxiety symptoms also indicate the importance of addressing individual's poor mental health symptoms in mitigating social isolation. Thus, results highlight that it is essential for future research not only to consider individual differences in social cognition (including ToM) when designing interventions to target social isolation in older adults, but also to account for and address co‐occurring mental health conditions when examining the social impact of autistic traits. Additionally, the potential bidirectional relationship between social isolation and poor mental health should be considered and explored in future longitudinal studies.

It is important to acknowledge limitations of the current study when contextualizing it. First, due to the study being targeted by spammers (an increasing problem across online studies; see, Roehl and Harland 2022), all responses were manually screened for spam indicators, and data from participants who failed quality control were excluded. While the methods used to screen participants were rigorous, stringent, and conducted by multiple members of the research team, it is possible that some ‘real’ participants were excluded, and ‘imposter’ participants included. Our study is not alone in facing this problem within autism research (i.e., see Pellicano et al. 2022), and robust methods to ensure the integrity of data are needed. Second, the number of participants in the present study was also slightly below the estimated sample size calculated using G*Power (123 participants were required to obtain 80% statistical power for detecting small to medium effects). This could result in our analyses not being statistically powered to detect small effect sizes (Nayak 2010; Faber and Fonseca 2014). As such, it is important that the findings of the current study are further replicated in larger samples, with sufficient statistical power to stratify samples into sub‐groups (e.g., non‐autistic and autistic groups, men and women) to examine whether the same mediating effect of ToM is found in groups with different neurotypes and gender identities. Third, the current study relies on self‐report and utilized a convenience sampling method; older adults that participate in research are usually healthier and functioning relatively better than those who do not (Golomb et al. 2012), and may potentially be less affected by social isolation. Future studies should employ larger samples ascertained through epidemiological sampling to minimize the risk of potential biases. Fourth, the results from the current study are cross‐sectional, thus causation cannot be inferred from our findings. Future studies employing a longitudinal design to assess changes in social isolation (with other related social, emotional, sensory and cognitive factors) over the lifetime, would be beneficial in building comprehensive understanding of the causes of social isolation. And finally, we did not ask participants whether they had previously engaged with any social skills training, which could result in strategies for better ToM ability while having high autistic traits; further research should examine the potential influence of this type of training when examining ToM ability (see Fletcher‐Watson et al. (2014) for systematic review of ToM training efficacy in autistic populations).

Whilst these limitations may affect the overall generalizability of our findings, results from the present study—which used established and standardized measures of autistic traits, social connectedness, and ToM tasks—still provide essential information that replicates the interrelationship between social connectedness, autistic traits, and ToM, along with increasing our understanding of ToM as one possible cognitive mechanism in the experience of social isolation in older adults with high autistic traits. Understanding potential underlying factors associated with social isolation could contribute to establishing future research in exploring prevention and protection mechanisms that would minimize the risk of social isolation in the aging population. Gaining a more profound understanding is crucial to building better support systems for older people's well‐being and reducing pressure on healthcare systems.

In conclusion, this study finds that high social connectedness in middle‐aged and older adults is associated with fewer autistic traits and better performance in ToM tasks. The importance of addressing ToM is further highlighted by its full mediating role when accounting for mental health factors. Our findings also underline the susceptibility of older autistic/high autistic trait people to becoming socially isolated, emphasizing the need to draw both clinical and research attention to the autistic older population's well‐being. Future longitudinal studies with large samples and measures of other related variables (e.g., household members, cognitive factors) are required to understand the underlying causes and mechanisms leading to social isolation in older age populations.

## Author Contributions

F.H. and G.R.S. conceived the current study. G.R.S. designed the online survey and selected materials. G.H.M.L., C.D., and G.R.S. conducted analyses. G.H.M.L. and G.R.S. wrote the manuscript, with F.H. reviewing and editing drafts. All authors have read and approved the final manuscript.

## Conflicts of Interest

The authors declare no conflicts of interest.

## Supporting information


Data S1.

## Data Availability

Research data are not shared.
