# Supplementary material for: Theory of Mind Mediates the Association Between Autistic Traits and Social Isolation in Middle‐Aged and Older Adults
Source: Autism Res. 2025 Apr 4;18(6):1234–44. doi: 10.1002/aur.70036 (PMC12166511; doi:10.1002/aur.70036)
Supplement: Supplementary file 1 — Data S1. [file AUR-18-1234-s001.docx]

**Supplementary Material A**

**Overview of Focus Group**

**Facilitated by:**

Dr Gavin R. Stewart

**Attendees:**

Person 1 (late diagnosed autistic woman, 70s)

Person 2 (late diagnosed autistic woman, 70s)

Person 3 (late diagnosed autistic woman, 70s)

Person 4 (late diagnosed autistic woman, 80s)

Person 5 (late diagnosed autistic man, 70s)

Person 6 (late diagnosed autistic man, 80s)

**Duration:**

2 hours

**Purpose and Aim:**

This focus group was hosted as part of the facilitator’s ESRC-funded Postdoctoral Research Fellowship. The aim of the focus group was to discuss issues related to ageing that were important to the attendees.

**Implementation:**

Using prompts developed in previous PPI/steering activities, the facilitator asked attendees about how various topics (e.g., health, cognition, social engagement) influence their quality of life.

The prompts used were open-ended, allowing for wider conversation between the attendees. A selection of the prompts related to social engagement were based on the following: “Some people find that their preferences for socialisation change as they get older, have you experienced this?”, “Has your ability to socialise with other people had an impact on your quality of life as you’ve got older, whether for good or bad?”, “Are there parts of socialisation that you find particularly helpful or challenging?”

**Outcome:**

The consensus of the focus group was that the attendees found their social networks began to shrink as they had grown older. Most noted that they were still very socially motivated and wanted friendships, but there were many challenges with maintaining friendships. Two attendees noted topics related to theory of mind (e.g., understanding what other people wanted from friendships was unclear, difficulties with balancing the intensity and back-and-forth of conversations, making small talk was off-putting). The facilitator discussed these concepts further with the focus group and some found they were good at these things while others found they struggled. This led to the facilitator suggesting the idea of examining the role of theory of mind in relation to autistic traits and social connectedness/isolation, which the attendees found interesting.

**Supplementary Material B**

**Example Stimuli for the CarToM Task (Livingston et al., 2023)**


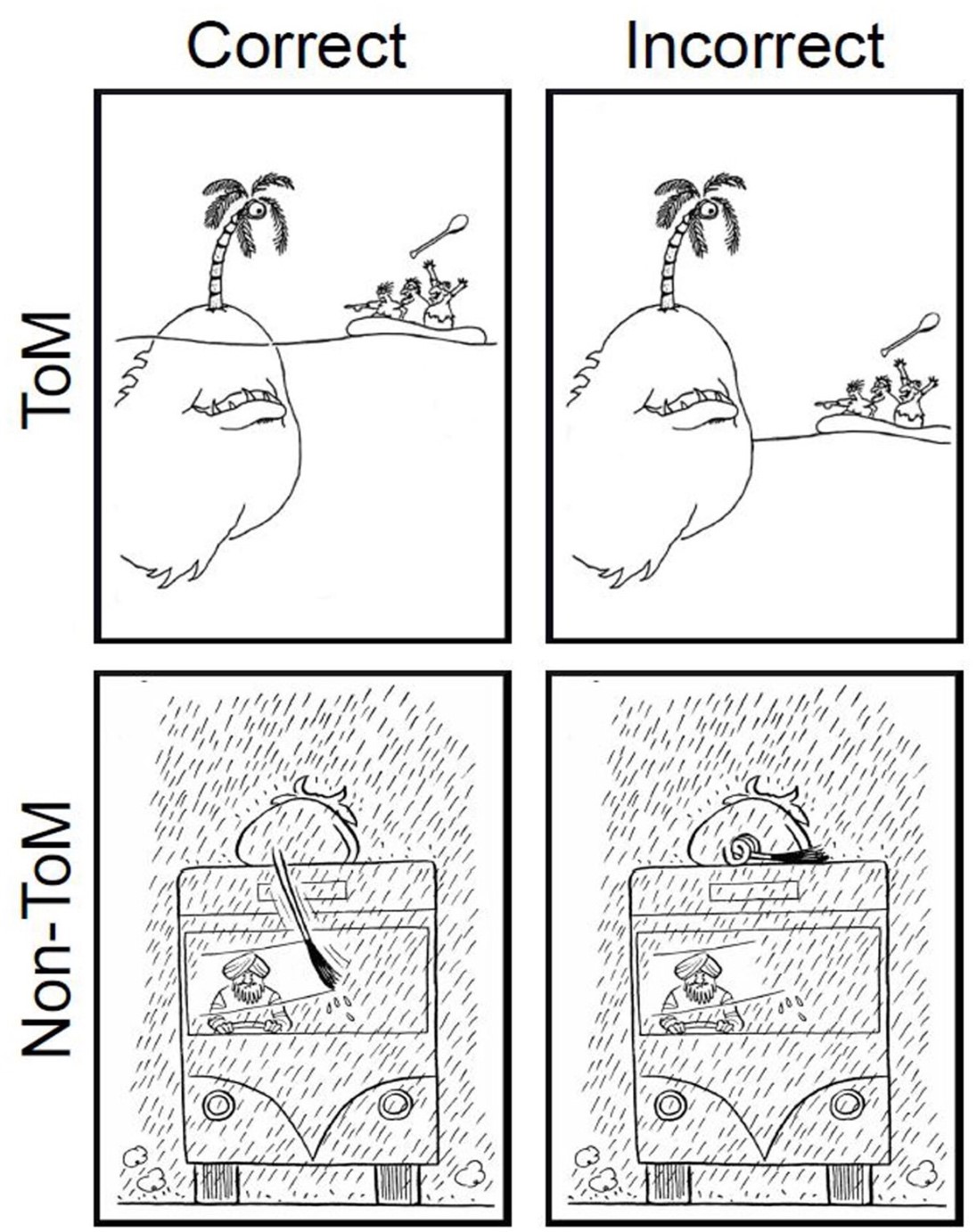


*Note.* Example cartoon pairs for the ToM condition (top) and non-ToM condition (bottom). The “original” humorous cartoons on the left side of each pair are the “correct” answer. Cartoon presented on the left are the edited, non-humorous version of the “original” humorous cartoons.

**Supplementary Material C**

**Covariate Controlled Correlations Matrices**

| *Table B1. Correlations Between Variables with Age Controlled* | | | | | | |
| --- | --- | --- | --- | --- | --- | --- |
| **Variables** | **Social**  **Connectedness** | **Autistic**  **Traits** | **CarToM LISAS** | **FHTA**  **Composite** | **Depressive Symptoms** | **Anxiety**  **Symptoms** |
|  |  |  |  |  |  |  |
| **Social Connectedness** | 1 |  |  |  |  |  |
| **Autistic Traits** | -.48*** | 1 |  |  |  |  |
| **CarToM LISAS** | -.51*** | .49*** | 1 |  |  |  |
| **FHTA Composite** | .54*** | -.59*** | -.44*** | 1 |  |  |
| **Depressive Symptoms** | -.43*** | .64*** | .39*** | -.26** | 1 |  |
| **Anxiety Symptoms** | -.45*** | .59*** | .44*** | -.29** | .82*** | 1 |
| *Note.* CarToM, Cartoon Theory of Mind; LISAS, Linear Integrated Speed – Accuracy Score; FHTA, Frith–Happé Triangles Animations. Higher CarToM LISAS = lower performance; Higher FHTA = better performance. ** p* < .05, *** p* < .01, **** p* < .001 | | | | | | |

| *Table B2. Correlations Between Variables with Age, Symptoms of Depression and Anxiety Controlled* | | | | |
| --- | --- | --- | --- | --- |
| **Variables** | **Social** | **Autistic** | **CarToM** | **FHTA** |
|  | **Connectedness** | **Traits** | **LISAS** | **Composite** |
| **Social Connectedness** | 1 |  |  |  |
| **Autistic Traits** | -.28** | 1 |  |  |
| **CarToM LISAS** | -.39*** | .32*** | 1 |  |
| **FHTA Composite** | .47*** | -.56*** | -.36*** | 1 |
| *Note.* CarToM, Cartoon Theory of Mind; LISAS, Linear Integrated Speed – Accuracy Score; FHTA, Frith–Happé Triangles Animations. Higher CarToM LISAS = lower performance; Higher FHTA = better performance. ** p* < .05, *** p* < .01, **** p* < .001 | | | | |

**Supplementary Material D**

**Simple Mediation Models Between Autistic Traits, ToM Task Performance and Social Connectedness While Controlling Age, Depressive and Anxiety Symptoms**

**Figure D1**

*Simple Mediation Model Between Autistic Traits, ToM Task Performance (CarToM LISAS) and Social Connectedness While Controlling Age, Depressive and Anxiety Symptoms*

*Note*. X = independent variable, M = mediating variable and Y = dependent variable. Path A = standardised regression coefficient representing the relationship between autistic traits and CarToM LISAS score. Path B = standardised regression coefficient representing the relationship between CarToM LISAS score on the level of social connectedness. Path C’ = standardised regression coefficient for a direct effect of autistic traits on level of social connectedness. Path C = standardised regression coefficient for the total effect of autistic traits on level of social connectedness through the mediator.

* p < .05, ** p < .01, *** p < .001

**Figure D2**

*Simple Mediation Model Between Autistic Traits, ToM Task Performance (FHTA Composite) and Social Connectedness While Controlling Age, Depressive and Anxiety Symptoms*


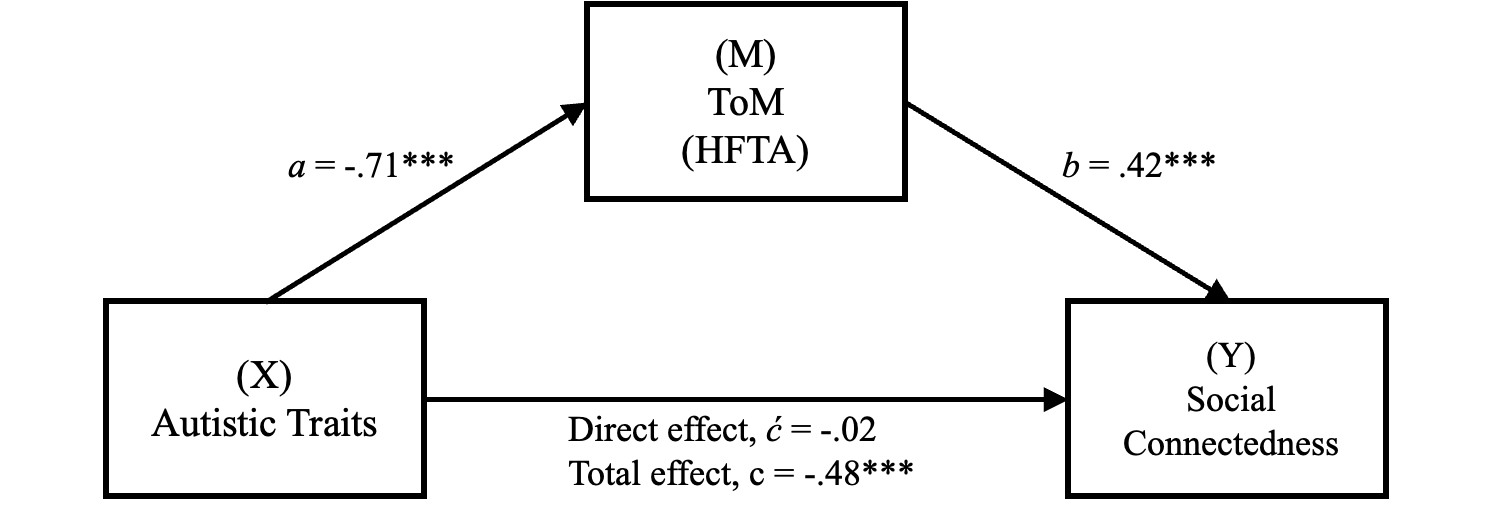
*Note.* X = independent variable, M = mediating variable and Y = dependent variable. Path A = standardised regression coefficient representing the relationship between autistic traits and FHTA composite score. Path B = standardised regression coefficient representing the relationship between FHTA composite score on the level of social connectedness. Path C’ = standardised regression coefficient for a direct effect of autistic traits on level of social connectedness. Path C = standardised regression coefficient for the total effect of autistic traits on level of social connectedness through the mediator.

**(FHTA)**

* p < .05, ** p < .01, *** p < .001
